# Therapeutic Uses of Retinol and Retinoid-Related Antioxidants

**DOI:** 10.3390/molecules30102191

**Published:** 2025-05-16

**Authors:** Janka Vašková, Marek Stupák, Martina Vidová Ugurbaş, Jozef Židzik, Helena Mičková

**Affiliations:** 1 Department of Medical Biology, Faculty of Medicine, Pavol Jozef Šafárik University, 040 11 Košice, Slovakia; janka.vaskova@upjs.sk (J.V.); helena.mickova@upjs.sk (H.M.); 2 Department of Medical and Clinical Biochemistry, Faculty of Medicine, Pavol Jozef Šafárik University, 040 11 Košice, Slovakia; 3 Second Department of Surgery, Faculty of Medicine, Pavol Jozef Šafárik University, 040 11 Košice, Slovakia

**Keywords:** antioxidant, carotene, carotenoid, oxo-carotenoid, polyene, proretinoid, retinoid, retinol, retinoic acid, retinal

## Abstract

Retinol and retinol-related compounds are essential for human health, particularly in cellular protection, skin health, and the management of medical conditions. Retinol—a vital form of vitamin A—is obtained through the diet as preformed vitamin A or provitamin A carotenoids, retinyl esters. These compounds are indispensable for vision, immune function, and skin health. While retinoic acid has important known biological roles, its presence is limited in the body as it is rapidly metabolized rather than stored, emphasizing the need for sufficient dietary intake. This paper is divided into chapters that highlight important aspects of retinol and retinoid-related compounds, such as their sufficient intake through food sources. The nutritional value of carotenoids is influenced by the balance between trans- and cis-isomers in food, with food processing affecting their bioactivity. Next, it is metabolism in the digestive tract. The bioavailability and efficacy of retinoids are further influenced by gut microbiota, which can modulate immune function and the expression of the genes involved in retinoid metabolism. A third important property greatly influencing their biological function is their structure, predisposing them to certain biological activities. Both retinoids and carotenoids exert key antioxidant functions by protecting cells from oxidative damage, quenching singlet oxygen, and stabilizing free radicals. However, the oxidation of carotenoids can result in various metabolites, such as epoxides and hydroxyketones, that further create a higher demand for antioxidant defenses. Additionally, carotenoids interact with lipoxygenases (LOXs), thus influencing oxidative stress, although this interaction may reduce their antioxidant efficacy. First- and second-generation retinoids regulate gene expression related to skin cell function and oncological diseases. Despite their therapeutic benefits, long-term use carries risks, such as teratogenicity. Ongoing research should aim to enhance the safety, precision, and effectiveness of retinoid therapies, expanding their therapeutic potential.

## 1. Introduction

Retinaldehyde (retinal) and all-trans-retinoic acid (ATRA) are physiological oxidative derivatives of retinol that represent physiologically active forms of vitamin A (vitamin A_1_). Another form, dehydroretinol (vitamin A_2_) contains another double bond between carbons 3 and 4 in the β-ionone ring. This yellow, fat-soluble vitamin intervenes in metabolism at various points in animal organisms. It is primarily an essential part of the biochemistry of visual perception and transcription control and activation, and it is an essential element for healthy embryonic development, differentiation, and proliferation [1] through its interaction with two kinds of nuclear receptors: retinoic acid receptors (RARs) and retinoid X receptors (RXRs) [2]. It is estimated that ATRA affects the activity of up to 500 genes and has anti-inflammatory, anti-cancer, and inhibitory effects on cell proliferation [3].

The major active form, ATRA, is formed from all-trans-retinol in two steps. First, retinol is oxidized into retinaldehyde by short-chain dehydrogenase/reductase (retinol dehydrogenase). Then, retinaldehyde is oxidized into ATRA in a reaction mediated by various retinaldehyde dehydrogenases. The oxidation of retinol into retinaldehyde is a reversible and rate-limiting step in the RA biosynthetic pathway, while the oxidation of retinaldehyde is irreversible and occurs at a higher rate than that of retinol [4,5]. Vitamin A was discovered in 1913 by the American biochemist Elmer McCollum and was first synthesized in 1947. Chemically, vitamin A contains a six-membered β-ionone ring in its molecule with a side chain comprising two isoprenoid units [6]. Chemically related analogs of vitamin A (derivatives of retinol and retinoic acid, monocyclic diterpenes) and its precursors, classified as retinoids, have similar properties.

Until 1980, retinol, all-trans-retinoic acid, and 9-cis-retinoic acid were considered biologically active. In addition, 4-oxo-retinol, 4-oxo-retinoic acid, and retinol metabolites retro-retinoids (14-hydroxy-4,14-retro-retinol, anhydroretinol) are also biologically active [7,8]. Retinoids can also be synthetically prepared derivatives used for specific purposes in treatment. Knowledge about the binding affinity of several other structures to retinoic acid receptors (RARs) has led to the redefinition of the term retinoids, now referring to substances of natural or synthetic origin that have a structure or activity similar to retinol [9].

**Figure 1:**
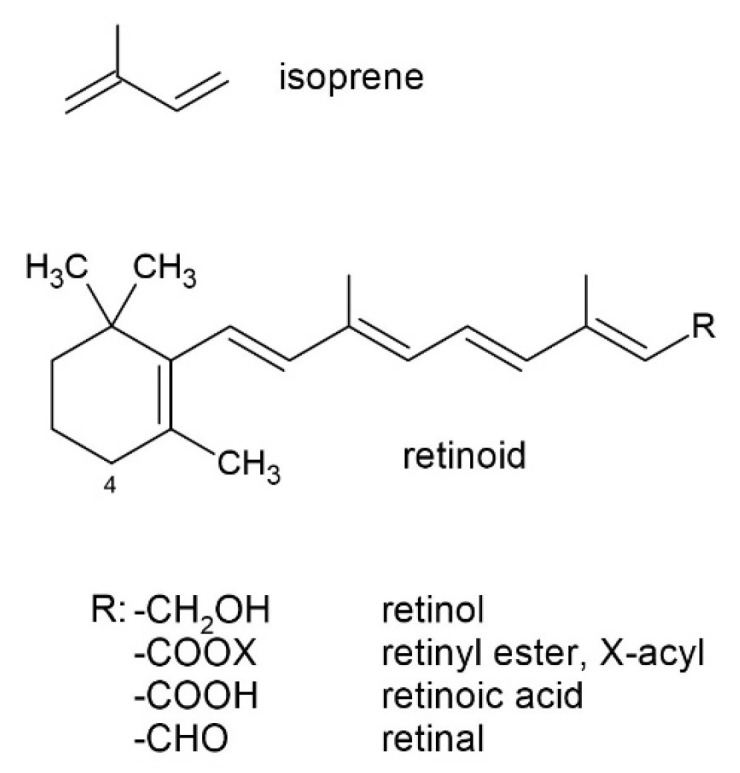
The general structure of a retinoid, where a polyene chain comprising two isoprene units (also classified as isoprenoids) and a β-ionone ring are present.

Retinol can be derived from the metabolic conversion of some dietary carotenoids, namely C40 isoprenoids (or tetraterpenoids). These carotenoids are called provitamin A carotenoids or proretinoids. To exhibit provitamin A activity, a carotenoid molecule must have at least one unsubstituted β-ionone ring and the correct number of methyl groups in the correct position in the polyene chain [10]. Only about 50 out of over 750 carotenoids are capable of metabolic conversion into the essential nutrient retinol (such as β-carotene, α-carotene, and β-cryptoxanthin) [11]. Naturally occurring retinoids are polyenes, as are first- and second-generation retinoids containing isoprene units (Figure 1). The chemical structure of polyenes is best represented by the formula R–(CH=CH)*_n_*–R, where *n* denotes the number of repeating units, and R can represent various capping groups [12].

Proretinoids, along with vitamins E, C, and D, are capable of the non-enzymatic regulation of reactive oxygen species (ROS) through the scavenging, quenching, inactivation, and termination of oxidative radical chain reactions, making them important antioxidant micronutrients not only for maintaining health but also in the context of various disease states [13,14]. The presence of multiple conjugated double bonds provides them with predominantly antioxidant properties (Figure 1). The hydrophobic chain of polyene units can quench singlet oxygen, neutralize thiyl radicals, and combine with and stabilize peroxyl radicals, corresponding to antioxidant activity. The longer the polyene chain, the greater the peroxyl radical stabilizing ability. Given their structures, vitamin A and carotenoids can autoxidize when O_2_ tension increases and, thus, are the most effective antioxidants at low oxygen tensions that are typical of physiological levels found in tissues [15].

The enhancement of antioxidant pathways has been observed, for instance, with vitamin A supplementation in autoimmune thyroiditis. Its antioxidant effects, combined with the modulation of the pituitary–thyroid axis, suggest a key role in promoting weight reduction and lowering body mass index [16]. Antioxidant properties, demonstrated by the elimination of reactive oxygen species (ROS), the restoration of antioxidant enzyme activities, increased glutathione levels, reduced malondialdehyde concentrations [17], and the prevention of advanced end glycation products formation [18], were observed in hyperoxia-induced kidney injury and hyperglycemia, respectively.

However, even in the area of the expected antioxidant properties according to the structure, there is an obvious need to take into account the action of retinoic acid through RAR/RXR receptors, as well as the level of post-translational modifications, which are also affected by the level of oxidation and carbonyl stress [19]. Therefore, it is necessary to mention the conflicting results of studies in reducing oxidative stress in patients with pancreatic disease [3] or even the role of oxidative stress in neural tube defect formation during early embryogenesis by exposure to high doses of retinoic acid [20].

This study summarizes retinoid metabolism in experimental mammals and focuses on the oxidative and antioxidant properties of retinoids, as well as the effects of first- and second-generation retinoids with structures resembling that of retinol.

## 2. Methods

To identify the relevant literature, multiple scientific databases were utilized, including PubMed, Scopus, Web of Science, ScienceDirect, and Google Scholar. The search was conducted from January 2024 to April 2025, ensuring the inclusion of the most recent findings. A comprehensive set of keywords was used—such as “retinol”, “retinoid”, “β-carotene”, “carotenoid”, “vitamin A”, “antioxidant”, “oxidation”, “oxidative stress”, “metabolism”, “digestion”, “resorption”, and “gut microbiota”—combined using Boolean operators (*AND*, *OR*), “first-generation retinoid”, and “second-generation retinoid” to refine and broaden the search. Both original experimental studies and review articles were considered. The initial screening involved evaluating the titles and abstracts for topical relevance. Full texts of shortlisted articles were then reviewed to assess the quality, depth of content, and relevance to the research focus. Priority was given to peer-reviewed sources, and articles were selected based on their scientific rigor, methodological transparency, and contribution to the understanding of retinoid biology. While the final selection reflected topical relevance and methodological quality, personal academic interest also guided the inclusion of specific articles.

**Figure 2:**
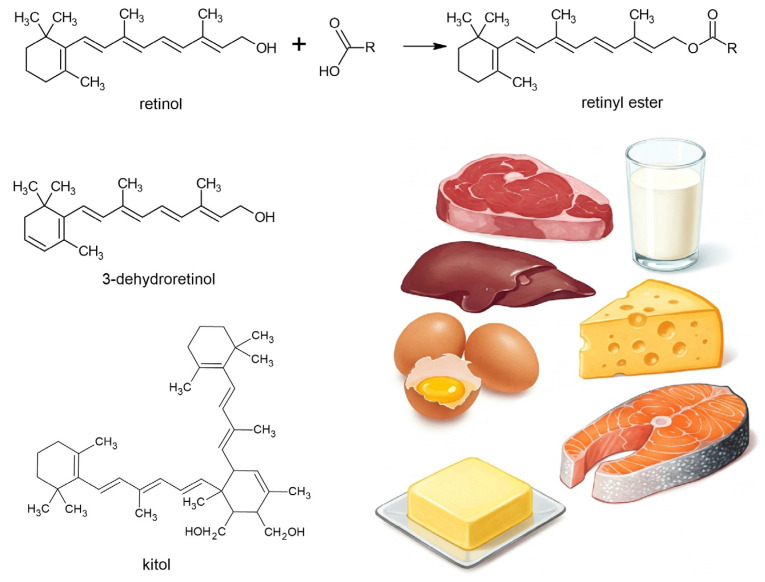
A general scheme for the esterification of retinol and illustrations of animal sources of retinoids and the structures of the most abundant ones.

**Figure 3:**
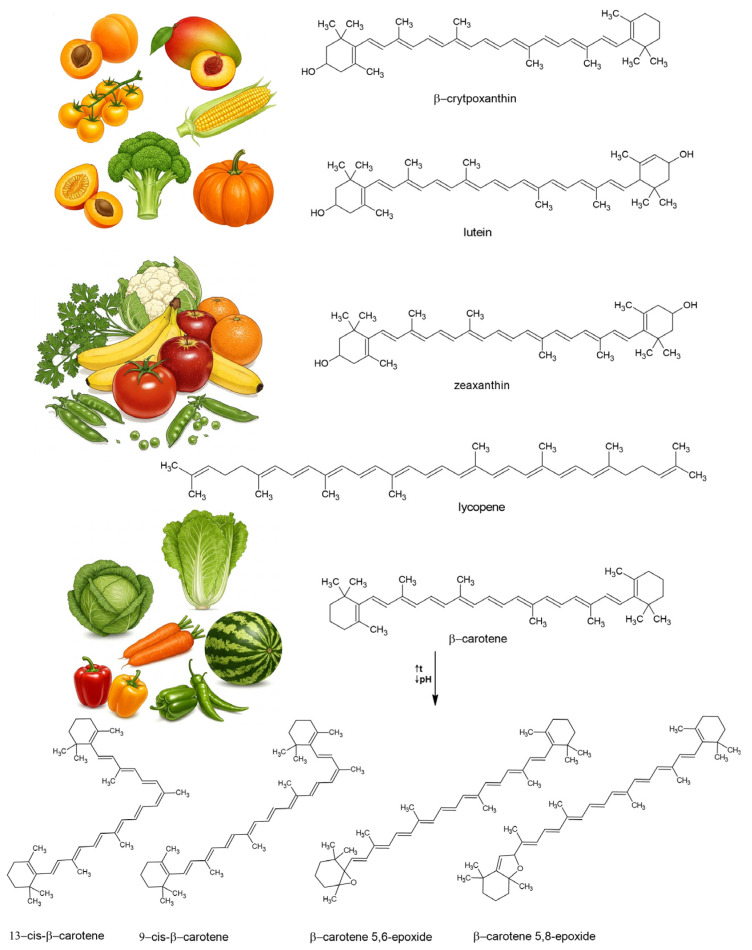
Illustration of plant sources of retinoids and the structures of the most abundant proretinoids and products of trans–cis-isomerization.

The image attachments included in Figure 2 and Figure 3 were created using Gemini AI, version 2.0 Flash.

## 3. Food Sources

Retinol is an essential vitamin and, therefore, it is necessary to obtain it from the diet either as preformed vitamin A or provitamin A carotenoids. Preformed retinol and retinyl esters are found in food of animal origin [21,22]. The main animal sources of retinol are liver, egg yolk, and milk fat [23]; that is, dairy products and butter (Figure 2). As free retinol is found in food only in low concentrations, it undergoes esterification with higher fatty acids (palmitic acid and stearic acid, but also lauric, arachidonic, linoleic, myristic, etc.). Therefore, it most often occurs in the form of retinyl esters. Retinyl acetate and retinyl palmitate are often used as food additives (butter, milk, cooking oil, and margarine). Retinyl esters are relatively stable in the absence of oxygen. A particularly rich source is cod liver oil. High levels of retinol are also found in beef and pork liver. Meat and milk are relatively low in vitamin A. Retinol and its derivatives isomerize to a mixture of products, where 13-cis and 9-cis stereoisomers predominate. ATRA occurs in food only in small amounts; in normal biological processes, it is less reactive than retinol or retinyl esters as it is not stored as a reserve but, instead, is intensively metabolized [24].

Naturally, most of the carotenoids occur as trans-isomers in plants. However, cis-isomers may increase due to the isomerization of trans-isomer carotenoids during food processing [25]. At higher temperatures or in light, carotenes can isomerize into various forms [26], exhibiting vitamin A activity only if at least one β-ionone ring is retained [27]; these are called neocarotenes. Lycopene is an acyclic carotenoid lacking a β-ionone ring and, therefore, has no provitamin A activity. Enzyme browning has also proven to be a basis for carotenoid isomerization [28]. The oxidative degradation of carotenoids also leads to trans–cis-isomerization and the formation of carotenoid epoxides [29]. According to Dugave and Demange [30], cis–trans-isomerization can occur in nine distinct ways, with four involving the formal breaking of the double bond through a homolytic or heterolytic process. These transformations are associated with changes in both physicochemical and biological properties, such as decreased provitamin A and antioxidant activity [25]. The main sources of proretinoids (Figure 3) are yellow and orange fruits and dark-green vegetables [31]. β-carotene shows higher provitamin A activity than β-cryptoxanthin and α-carotene [32]. Trans- and cis-isomers have been detected in some fruits and vegetables; for example, by Khoo et al. [26], Dzakovich et al. [33], and Saini et al. [34].

In Western countries, more than 70% of the daily vitamin A intake is derived from preformed vitamin A found in animal sources, while less than 30% comes from provitamin A carotenoids in plant-based foods. In contrast, in developing countries, over 70% of daily vitamin A intake comes from provitamin A carotenoids found in fruits and vegetables [35]. The minimum daily intake (μg retinol equivalents [μg RE]) necessary to prevent xerophthalmia varies according to age, from 180 to 450 μg RE/day [9]. In addition, other factors, such as gender and body weight, should be considered. The World Health Organization (WHO) has provided a list of estimated mean vitamin A requirements for different populations. These estimates are valuable for determining the Recommended Dietary Allowance (RDA) for specific population groups [9]. Deficiency can lead to nyctalopia [36] and is associated with an increased risk of various chronic diseases, including cardiovascular disease, cancer, and immune system disorders [37]. The use of natural carotenoids as food colorants is common, such as in the European Union and the United States [38]. Several commercially prepared derivatives are produced and used as nutritional supplements or food fortifiers. These derivatives include retinyl acetate [39], retinyl propionate for feed and liquid premix applications, and retinyl palmitate for human nutrition [40]. Although retinol is essential, an intake of 3000 ugRAE/day is associated with toxicity [41].

## 4. Overview of Digestion, Absorption, and Distribution into Tissues

**Figure 4:**
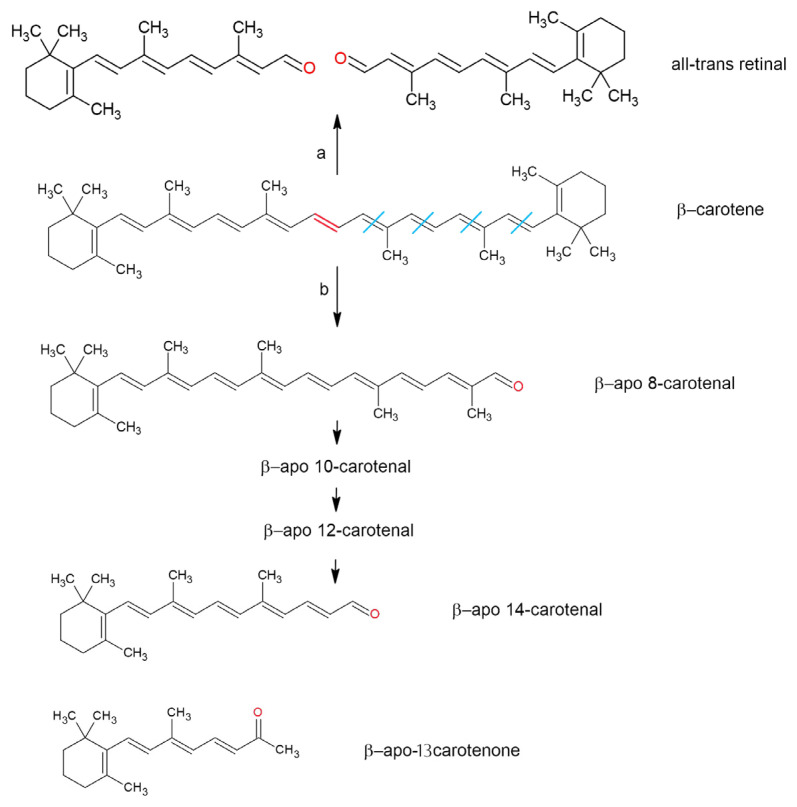
The oxidation of the double bonds of β-carotene on random sites can produce various molecules. (a) Two molecules of retinal are formed. The reaction also occurs under the catalysis of β-carotene-15,15′-dioxygenase, and (b) oxo-carotenoids of different chain lengths are formed.

As lipid-soluble molecules, retinoids and carotenoids are linked to lipid digestion. Primarily, they are found in plant and animal cell membranes and lipid droplets and are released from their food matrices alongside other lipid constituents during digestion [42]. Solubilization in the lipid phase and incorporation into micelles make them accessible for uptake in the duodenum [43]. However, the overall yield of this process is influenced by various factors, and only a small fraction of consumed carotenoids is absorbed during digestion [44]. ATRA is found in food only in small amounts, mainly in the form of retinyl esters. Upon ingestion, the all-trans-isomer of β-carotene is preferentially absorbed over its cis-isomers in humans [45,46]; however, in the ferret model [47], all-trans-β-carotene is more bioavailable than 9-cis- and 13-cis-β-carotene in gerbils [48]. Due to their structural similarity, the cleavage products of β-carotene (Figure 4) are metabolized by some of the same enzymes and pathways involved in metabolizing retinal, retinol, and retinoic acid [11,49].

### 4.1. Oral Cavity

Sensitization to lipids in the oral cavity is the initial step in their digestion. Lai et al. [50] demonstrated lipolytic activity in approximately 45% of their study participants but without identifying its sources. The small number of fatty acids released from triacylglycerols by lipolysis bind to receptors in human taste buds, where they act as signaling molecules [51]. The effect of lipolytic activity in the oral cavity on vitamin esters and carotenoids is not yet known, but carotenoids are present in saliva [52]. Blakeley et al. [53] showed that saliva contains very low levels (2 μg/mL) of retinol-binding protein (RBP). The role of RBP in saliva is unclear, but it may be related to the micellar absorption of the vitamin. Some proteins in food, such as β-lactoglobulin, can bind β-carotene and retinol [54], as well as α- and β-caseins, forming a complex with retinol [55]. β-lactoglobulin partially resists gastric digestion [56] and is slowly hydrolyzed only in the duodenum [57], so some β-lactoglobulin may reach enterocytes. Animal feed, food, and fortified food products contain only a small fraction of free retinol; instead, they mainly contain retinyl esters, which show virtually no affinity for RBP [58]. Since free retinol is labile under acidic conditions [59], salivary RBP may protect free retinol in food from degradation in the stomach. Low et al. [60] demonstrated that human mastication can lead to a 35% increase in the release of β-carotene from the plant matrix during in vitro gastric and intestinal digestion.

### 4.2. Stomach

Some carotenoids, as well as fat-soluble vitamins, are transported by lipid droplets within the stomach [61]. The size of the droplets does not affect the efficiency of vitamin A absorption in healthy humans, and no vitamin A degradation or absorption occurs at the stomach level [61]. Gastric lipolysis contributes to approximately 25% of the digestion of triacylglycerols and activates pancreatic lipase in certain lipid substrates [62], with a significant impact on the hydrolysis of carotenoid esters. Emulsions with smaller droplet diameters (0.2 µm vs. 23 µm) can enhance the transfer of β-carotene from lipid droplets to mixed micelles, increasing its bioaccessibility from approximately 35% to 60% [63]. However, the incorporation of carotenoids into gastric emulsion is influenced by several limiting factors, including soluble proteins, surface charges in the gastric emulsion, oils, and the number of carotenoids present [64]. For example, the incorporation of β-carotene into the gastric emulsion is inhibited by soluble proteins that affect the interfacial characteristics of the digesta. Qiu et al. [65] found that gliadin reduces the enzymatic degradation of lipids and prevents digestive enzymes from adsorbing to the droplet surface or directly binding to enzymes, thereby negatively affecting micelle formation. However, proteins, such as caseins, are highly surface-active molecules, and the formed particles tend to be highly negatively charged, preventing lipid droplet aggregation and actually stabilizing emulsions in the GI tract after adsorption to lipid droplet surfaces [42]. Whey protein isolates inhibit lipid oxidation and promote the formation of smaller lipid droplets, enhancing the bioavailability of β-carotene [66].

Another factor is pH. At a low pH, the concentration of soluble proteins decreases, and the transfer of β-carotene to oil increases [64]. The solubilization of β-carotene increases as the surface charge of the gastric emulsion decreases [64]. The acidic pH (3–5) of the stomach can lead to small losses of β-carotene, which initially forms carotenoid cations [67] and can subsequently lead to trans–cis-isomerization. However, according to in vitro studies by Failla et al. [68] and Ferruzzi et al. [69], significant isomerization does not occur. Oxidizing agents such as iron, in turn, increase the formation of carotenoid oxidation products, such as β-apo-carotenals, epoxides, and other cleavage derivatives [70,71,72]. However, in human digestive conditions, this has only been confirmed to a very limited extent [73].

### 4.3. Intestinum

The free forms of vitamin A and carotenoids are absorbed by the intestinal mucosa [74]. Dietary retinol is taken up directly by mucosal cells. However, dietary retinyl esters cannot enter the intestinal mucosa [75]. All enzymes produced by the pancreas hydrolyze the food matrix within the intestinal lumen and promote the release of retinyl esters [76]. The luminal hydrolysis of retinyl esters occurs through the action of pancreatic lipase (LP) with pancreatic lipase-related protein 2 [77]. Carboxyl ester lipase (CEL) has been found to hydrolyze esterified carotenoids [78]. Esters that are not hydrolyzed by LP or CEL can be cleaved by brush border membrane retinyl ester hydrolase [79]. Finally, some esters may be absorbed intact by intestinal cells and hydrolyzed intracellularly [80].

Bile acids and salts in the small intestine enable the formation of mixed micelles of about 3–8 nm in diameter [64,81], which maintain the solubility of lipophilic compounds [82]. A higher concentration of lipase and bile promotes micellization [83,84]. Increasing dietary fat enhances micelle formation up to an optimal threshold [85], and longer fatty acyl chains promote more extensive micelle formation [64]. Proteins can aid in emulsification [86], but they can also have the opposite effect [34]. Fiber and high levels of minerals can hinder the formation of micelles [64,87]. Furthermore, carotenoids and retinol are absorbed if they are present in mixed micelles [81], although some may be incorporated into vesicles and liposomes within the same aqueous fraction. Retinol can be incorporated into phospholipid bilayers [88,89], where it enhances vesicle stability against bile salt deoxycholate [88]. Apolar carotenoids (such as β-carotene) accumulate in the core of the mixed micelle, which passes the mucus layer to the unstirred water layer of the enterocytes [90]. As Reboul concluded [91], the absorption of retinol varies by 75–100%, and that of β-carotene ranges between 3 and 90%.

Retinol enters intestinal cells through simple diffusion within a range of approximately 0.5 to 130 µM [92]. It is specifically transported by the protein STRA6 (STimulated by Retinoic Acid 6), which acts as a receptor for retinol-binding protein (RBP) [93]. STRA6 is believed to facilitate the uptake of both micellar retinol and retinol bound to β-lactoglobulin, functioning as a bidirectional transporter [94]. Retinol-binding protein 2 (RBP2, formerly known as cellular retinol-binding protein, type II (CRBPII)) binds to retinol or retinaldehyde in adults, facilitating retinoid uptake and metabolism within the intestinal epithelium [95]. In mammals, several retinoid-binding proteins mediate the transport and metabolism of retinoids in various tissues and organs, as summarized by Blaner et al. [96]. Carotenoid uptake is mediated by lipid transporters, such as scavenger receptor class B type I (SR-BI), in the membrane of enterocytes from the duodenum to the colon [97]; Cluster Determinant 36 (CD36) [98]; and Niemann–Pick C1-Like 1 protein (NPC1L1) [99]. However, these transporters are also found in several other tissues [91].

Proretinoids can be converted into retinal within enterocytes by β-carotene-15,15′-dioxygenase (BCMO1) [100]. RBP2 is present at high concentrations in enterocytes and binds both retinal and retinol [101]. Retinal reduces into retinol through an intestinal retinal reductase [102]. Proretinoid carotenoids can also be cleaved, together with non-provitamin A carotenoids, into apocarotenoids by mitochondrial β-carotene-9′,10′-dioxygenase (BCDO2) [103]. Retinol bound to RBPII is esterified into retinyl esters by lecithin retinol acyl transferase (LRAT) and acyl-CoA acyl transferase (ARAT) [104]. Furthermore, retinyl esters and dietary lipids packed in nascent chylomicrons are secreted into the lymphatic system and, subsequently, the bloodstream.

Many studies point to an important role of gut microflora in retinoid metabolism affecting health through the modulation of immune function, lipid metabolism, and other nutrient metabolism. It was shown that supplementation with vitamins such as C and E, including beta-carotene, can benefit the host by supporting normal intestinal barrier function and modulating the immune system [105]. Pham et al. [106] summarized studies suggesting that vitamin A influences microbial composition, with some reports indicating that an adequate vitamin A status may be associated with increased microbial diversity. For example, retinoid signaling, synthesis, transport, and concentration are significantly reduced in patients with atopic dermatitis. The modification of the gut microbiome with prebiotics and probiotics leads to the increased production of short-chain fatty acids, the activation of the retinol metabolic pathway in Peyer’s patches of the small intestine, and the modulation of the expression of genes for inflammatory cytokines and their receptors. This mechanism may contribute to an increase in the proportion of immature dendritic cells and regulatory T lymphocytes, thereby promoting the establishment of immune tolerance [107]. A study of Han et al. [108] showed ATRA supplementation increased microbial diversity and induced the growth of beneficial bacteria, such as *Parabacteroides*, *Bacteroides*, *Clostridium_XVIII*, *Bifidobacterium*, *Enterococcus*, *Bacillus*, *Leuconostoc,* and *Lactobacillus*, in obese mice. The gut microbiota modulated the expression of genes involved in retinol and lipid metabolism, leading to a reduction of body weight and the decreased accumulation of white adipose tissue in mice.

### 4.4. Liver

Chylomicron residues can diffuse into the hepatocytes of the liver. As mentioned in [109], 66–75% of retinyl esters are transported to the liver as chylomicrons. In the liver, hepatocytes hydrolyze retinyl esters into free retinol, which binds to RBP4 [110]. Proretinoids also bind to RBP4, and this complex with RBP reaches the bloodstream and target cells. Binding to RBPs serves as a selective mechanism for the specific oxidation of retinol by retinol dehydrogenase. This enzyme exhibits a higher affinity for the retinol–RBP complex than non-specific alcohol dehydrogenase [111,112].

In the liver, free retinol is esterified by LRAT and is stored in non-parenchymal hepatic stellate cells. LRAT uses phosphatidylcholine as fatty acid donors, mainly palmitic and, to a lesser extent, stearic and oleic acid [113,114,115]. Although hepatocytes play a key role in retinol uptake and mobilization, they contain only 10–20% of the total retinoid present in the liver; the remainder can be found in non-parenchymal hepatic stellate cells [116]. The liver can secrete retinyl esters bound to VLDL into the circulation. During VLDL metabolism, some retinyl esters may be transferred to LDL or incorporated into HDL [109].

Excess unesterified retinol is degraded by cytochrome P450 into various metabolites that have specific uses in different organs. In liver microsomes, ATRA undergoes biotransformation via cytochrome P450 (CYP26) through hydroxylation and oxidation, generating various metabolites. Hydroxylation produces 4-hydroxy-retinoic acid, a product that no longer has biological or pharmacological effects, which is further oxidized into 4-oxo-retinoic acid. These lipophilic acids are detoxified and eliminated via conjugation with glucuronic acid, forming glucuronides. The intracellular concentration of ATRA is regulated not only by its rate of synthesis but also by its conjugation with glucuronic acid and subsequent elimination. Retinoyl-β-glucuronide is by far the most abundant product of UDP-glucuronosyltransferase, which can even act as a non-toxic substitute for ATRA at higher doses [117]. Cytochrome P450 can also co-oxidize ATRA with lipid hydroperoxides, a process that critically depends on the availability of the cofactor NADPH. Additionally, CYP can utilize lipid hydroperoxides generated by prostaglandin synthase. Co-oxidation with arachidonic acid serves as a significant alternative pathway for ATRA inactivation [118].

### 4.5. Bloodstream

In the bloodstream, chylomicrons are degraded (triacylglycerols are released), with chylomicron residues rich in retinyl esters standing out. In chylomicrons, retinyl esters undergo lipolytic degradation via lipoprotein lipase, resulting in their hydrolysis into retinol. The released retinol then binds to cellular RBP1 in tissues, facilitating its absorption. In rodents, approximately 25–33% of chylomicron-derived retinyl esters are directly delivered to peripheral tissues [109].

Retinol bound to RBP4 in the retinol–RBP4–transthyretin complex is taken in by cells [119] via STRA6 [120,121,122]. Retinyl esters bound to LDL particles, as well as to albumin and retinyl-/retinoyl-β-glucuronides, may also be taken up into peripheral tissues [102].

The metabolism of retinoids in the digestive system plays a key role in their bioavailability, transport form, and subsequent distribution to target tissues, where they perform their functions and have therapeutic potential. Given the long-standing interest and intensive study of the metabolism and effects of retinoids, we would like to mention several studies devoted to these biological activities [123,124,125,126,127,128,129].

## 5. Antioxidant Properties and Oxidation of Retinoid-Related Compounds

Antioxidants prevent or slow down the damage to cells caused by ROS produced as byproducts of normal cellular metabolism or from external sources like pollution, radiation, or even dietary sources. ROS can cause oxidative stress, which contributes to aging and various diseases such as cancer, heart disease, and neurodegenerative disorders. Antioxidants neutralize ROS by donating electrons, thus stabilizing them and preventing further cellular damage.

Retinoids and proretinoids protect cellular components from damage caused by photo-oxidation and ROS through multiple mechanisms:

(a) A high molar absorption coefficient, enabling protection against photo-oxidation;
(b) Their ability to quench singlet oxygen (^1^O_2_);
(c) Their capacity to lose protons upon interacting with reactive species, forming a less reactive radical center stabilized by the polyene network [130].

The initial species that react with singlet oxygen are reactive dioxetanes, peroxides, epoxides, and endoperoxides, which can undergo secondary reactions. Various cis–trans, stereo-, and regio-isomers are formed [131]. As discussed in Washington et al. [131], retinoic acid can form nine products with ^1^O_2_: epoxide, furan, endoperoxide, hydroxyketone, dioxetane, and four degradation products (products with molecular weights lower than that of the parent retinoic acid). Similar oxidation products have also been reported regarding the oxidation of retinal, retinol, and retinol palmitate. Bisretinoid isolated from human lipofuscin reacts with singlet oxygen to form the polyepoxide species [132].

Retinoids (carotenoids themselves) are prone to one-electron oxidation due to conjugated polyene chains, forming carotenoid cationic radicals. The carbon atom in the 4-position (Figure 1) is highly reactive because it is allylic to the 5,6-double bond of the β-ring system, which is fully conjugated with side-chain double bonds. As a result, both carbocation and carbon-centered radicals that form in the allylic 4-position are readily stabilized through delocalization [133,134]. The radical at C-4 can combine with molecular oxygen to form peroxyl radicals (R-O-O^•^) [118], which form epoxides (5,6- and 5,8-epoxides). Peroxyl radicals also undergo direct reduction through the direct abstraction of a hydrogen atom from nearby macromolecules. The reduction potentials of carotenoid cationic radicals fall within a similar range of 1020 ± 40 mV. The relative ease of electron transfer to the carotenoids follows the order astaxanthin > canthaxanthin > zeaxanthin > β-carotene > lycopene [135]. The presence of carbonyl groups increases the reduction potential of carotenoids [136,137]. Oxo-carotenoids (with conjugated carbonyl groups, e.g., canthaxanthin) are electron-deficient due to their conjugated carbonyl groups, and the resulting carotenoid cationic radicals are destabilized. Consequently, the loss of a H^•^ or a single electron from polyene is unlikely to be the primary mechanism of antioxidant activity. Instead, anionic or radical reactive oxygen species, such as the superoxide radical (O_2_^•−^) or ^1^O_2_, readily transfer a single electron, thus forming resonance-stabilized oxo-carotenoid anionic radicals [138,139].

Hydrophobic lipid peroxyl radicals specifically interact with carotenoids, similar to their interaction with polyunsaturated fatty acids, resulting in oxidation products [140]. The oxidation of conjugated double bonds by reactive oxygen species also gives rise to numerous compounds, which is especially important in terms of localization in tissues and organs and their eventual elimination, e.g., 5,8-endoperoxy-2,3-dihydro-β-apocarotene-13-one, 3-hydro-4-oxo-7,8-dihydro-β-ionone, and 3-hydroxy-4-oxo-β-ionone [141,142,143,144]. Eight carotenoid metabolites and oxidation products, including anhydrolutein and 2,6-cyclolycopene-1,5-diol, have been detected in human plasma [145].

These mechanisms (also partly described by Kiokias et al. [146]), can be summarized for β-carotene as follows:

^1^O_2_ + ^3^β-carotene* → ^3^O_2_ + ^3^β-carotene* (1)

^3^O_2_ + ^3^β-carotene* → β-carotene-endoperoxide (2)

^3^β-carotene* → O_2_^•−^ + β-carotene^•+^ (3)

^3^β-carotene* → β-carotene + heat (4)

O_2_^•−^ + β-carotene → β-carotene^•−^ (5)

R-O-O^•^ + β-carotene → R-O-O-β-carotene^•^ (6)

R-O-O-β-carotene^•^ + R-O-O^•^ → inactive products (7)

R-O-O-β-carotene^•^ → R-O^•^ + β-carotene-epoxide (8)

R-O-O-β-carotene^•^ + O_2_ → R-O-O-carotene-O-O^•^ (9)

Reactions lead to the formation of various metabolites, such as β-carotene epoxides, endoperoxides, and peroxyl radicals. While the reactions produce some inactive products, others can lead to the breakdown of β-carotene into more reactive species, further contributing to oxidative damage.

The oxidation of conjugated double bonds in β-carotene at random sites—along with eccentric cleavage at double bonds other than the central one (which can also be catalyzed by β-carotene 9′10′-dioxygenase 2)—forms aldehydes, retinal, and β-apocarotenals of varying chain lengths (Figure 4). These are further cleaved into shorter-chain compounds or undergo β-oxidation to form retinoic acid [147]. β-apocarotenals are reduced into the corresponding β-apocarotenols by aldehyde reductases and/or alcohol dehydrogenases. β-apocarotenals can also be oxidized into the corresponding β-apocarotenoic acids by aldehyde dehydrogenases [11]. Research suggests that β-apocarotenoids formed under oxidative stress can interfere with nuclear receptor signaling. Notably, β-apo-13-carotenone acts as a high-affinity antagonist for all three retinoic acid receptors (RARα, RARβ, and RARγ) [148].

Lipoxygenases (LOXs) catalyze the oxidation of polyunsaturated fatty acids containing at least one 1Z, 4Z-pentadiene moiety, producing hydroperoxides. Additionally, some LOXs can co-oxidize carotenoids [149]. The initial step of the LOX reaction involves removing a hydrogen atom from a methylene unit between double bonds in the substrate fatty acids. The resulting carbon radical is stabilized through electron delocalization across the double bonds. Next, molecular oxygen is added to the carbon atom at the +2 or −2 position relative to the original radical carbon, forming a peroxyl radical and a conjugated trans–cis diene chromophore. The peroxyl radical is then hydrogenated to produce a hydroperoxide. Both the initial hydrogen removal and subsequent oxygen addition occur on opposite (or antarafacial) sides relative to the plane formed by the 1Z, 4Z-pentadiene unit [150]. The co-oxidation of carotenoids by LOX forms hydroperoxides, reducing their antioxidant potential [149]. Goldreich et al. [151] demonstrated that retinoids (such as all-trans-retinol, ATRA, and 13-cis-retinoic acid) can bind to the active sites of LOX1 and LOX2 or, alternatively, act as antioxidants simultaneously. Through models, Hazai et al. [152] found that among the six functional LOXs in humans, lycopene and lycophyll have a high affinity to bind in the cleavage site of 5-LOX, potentially leading to the direct competitive inhibition of 5-LOX activity after the in vivo supplementation of carotenoids. This ability to interact was also confirmed in a study by Lockwood et al. [153]. Therefore, β-carotene is in the spotlight for research into mechanisms in food processing that prevent LOX-mediated oxidation, rancidity, and flavor changes. Lutein, zeaxanthin, and β-cryptoxanthin, in turn, inhibit LOX and enhance the antioxidant potential of retinoids [149].

Prostaglandin H synthase (PGHS) catalyzes the biotransformation of ATRA. Lipid hydroperoxides generated by the cyclooxygenase activity of PGHS oxidize retinoids into carbon-centered radicals. The subsequent addition of molecular oxygen forms peroxyl radicals, which mediate the conversion of additional ATRA molecules into corresponding 5,6-epoxide, 5,8-epoxide, and related products [154]. ATRA is co-oxidized during the reduction of lipid hydroperoxides into their corresponding lipid hydroxides. Both ATRA and 13-cis-RA undergo hydrogen atom abstraction from the carbon at position 4 of the β-ionone ring system, forming radicals centered on the central carbon [134]. Nadin and Murray [118] demonstrated that PGHS and CYP contribute similarly to the oxidation of ATRA in human liver fractions.

## 6. Therapeutic Uses of First- and Second-Generation Retinoids

### 6.1. First-Generation Retinoids

**Figure 5:**
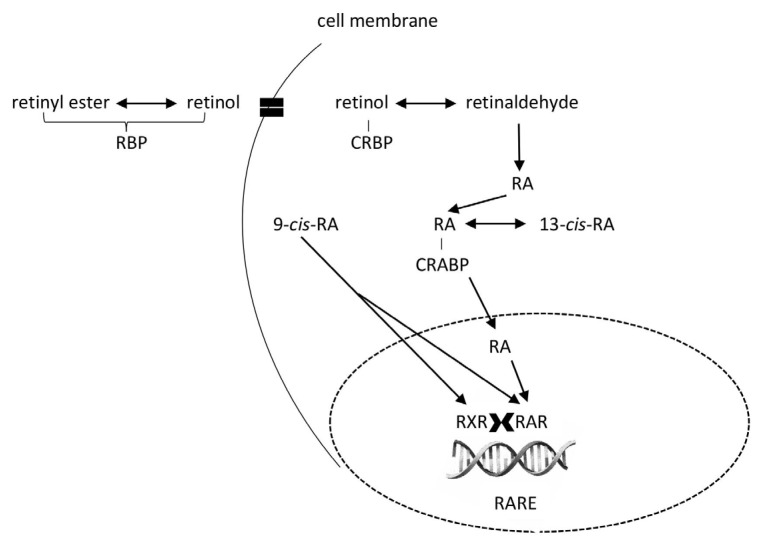
A schematic representation of the action of first-generation retinoids (tretinoin—ATRA (RA), isotretinoin—13-cis-retinoic acid (13-cis-RA), and alitretinoin—9-cis-retinoic acid (9-cis-RA)). Retinyl esters and retinol are transported to tissues bound to retinol-binding protein (RBP) and enter cells via the STRA6 (STimulated by Retinoic Acid 6) receptor. Through retinyl ester hydrolase, retinyl esters are converted to retinol, which is bound to the cellular form of RBP (CRBP). Retinol is converted through retinaldehyde to ATRA. In addition, 13-cis-RA, and 9-cis-RA can undergo isomerisation to ATRA. RA, 13-cis-RA, and 9-cis-RA bind to RA receptors (RAR) and retinoid X receptors (RXR) in the cell nucleus. These receptors then form heterodimers that bind to the RA response element (RARE), thereby activating the transcription of target genes.

Tretinoin (all-trans-retinoic acid), isotretinoin (13-cis-retinoic acid), and alitretinoin (9-cis-retinoic acid) are first-generation retinoids. Retinoic acid binds to retinoic acid receptor α (RARα), a member of the nuclear receptor superfamily, which also includes steroid hormone and thyroid hormone receptors. RARα forms heterodimers with the retinoid X receptor (RXR), increasing its specificity and ability to regulate gene expression. This complex binds to retinoic acid response elements (RAREs), which are present in the promoter regions of genes responsible for cell differentiation, proliferation, and apoptosis (Figure 5). RARα activation plays an important role in tissue development and regeneration, particularly in embryogenesis, the immune system, and the maintenance of epithelial structures [155].

Evidence suggests that the mechanism of action of topical tretinoin is mediated by binding to retinoic acid receptors (RARs) α, β, and γ, but this appears to lead to effective gene expression only via RARβ and RARγ [156] and RXRs. This interaction blocks inflammatory mediators, thereby reducing the inflammatory response in the skin [157]. Procollagen production increases, promoting the formation of collagen types I and III, improving the structure and firmness of the skin. This leads to skin regeneration, rejuvenation, and wrinkle reduction. The effects of RARγ are primarily associated with mucocutaneous tissues and bones, playing an important role in maintaining epithelial cell homeostasis. As an effective treatment for acne, tretinoin modifies the abnormal follicular keratinization of epithelial cells. This mechanism enables the detachment of cornified cells and increases shedding, thereby preventing pore blockage and stimulating mitotic activity. This accelerates cell turnover and eliminates loosely adherent corneocytes (comedolysis), thereby reducing the formation of microcomedones [158]. Tretinoin may help reduce epidermal melanin and pigmentation by increasing keratinocyte turnover and inhibiting tyrosinase activity [156]. As detailed by Sitohang et al. [159] and Mambwe et al. [160], numerous studies have investigated the effectiveness of tretinoin in treating and slowing the progression of photoaging, all of which have reported positive results. Recently, a tretinoin-loaded, nanostructured, lipid-carrier-based sunscreen to enhance tretinoin’s photostability and reduce its skin irritation was developed. The optimized formulation demonstrated improved stability, extended release, and enhanced UV protection, indicating the nanostructured lipid carrier as a promising carrier for topical tretinoin delivery [161]. RARα and RARβ are associated with acute promyelocytic leukemia (APL) and squamous cell malignancies, where they play a role in regulating cell proliferation and differentiation [158].

**Figure 6:**
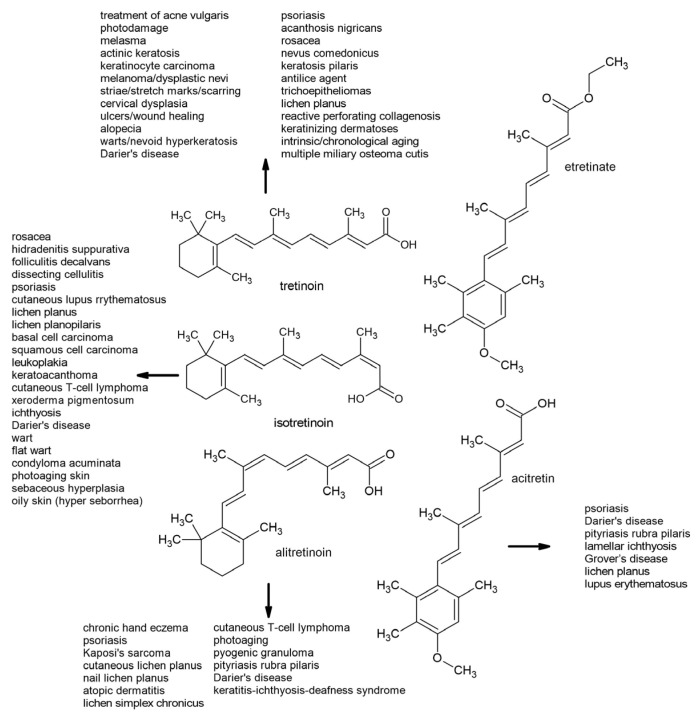
A depiction of the structures and usefulness of first- and second-generation retinoids in the treatment of various diseases (according to Baldwin et al. [156], Kaushik and Lebwohl [185], Paichitrojjana and Paichitrojjana [186], and Bubna [187]).

Isotretinoin is an orally administered systemic retinoid that has proven effective in treating acne at doses ranging from 0.5 to 1.0 mg/kg/day. While the precise mechanism of action remains unclear, isotretinoin at pharmacological doses inhibits sebaceous gland activity and keratinization. This drug has been shown to decrease the size of sebaceous glands and reduce sebum production, though there may be differences in guidelines and the consensus regarding the treatment of patients with acne (Figure 6). The cumulative dose over the entire course of treatment is a significant factor in preventing relapse [162,163]. Patients younger than 20 years of age, those with macrocomedone acne, and those with persistent lesions after treatment are more likely to relapse. The median time to relapse is ten months [164]. In the treatment of neuroblastoma, medical research and clinical experience have shown that isotretinoin can reduce cell proliferation and promote cellular differentiation [165,166]. It demonstrates anti-inflammatory and immunomodulatory properties by reducing monocyte TLR-2 expression, minimizing the inflammatory cytokine response, and exhibiting antineoplastic effects, making it a valuable treatment option for various skin diseases [167,168].

Alitretinoin binds to all RAR and RXR subclasses. This binding triggers a series of processes that ultimately result in the expression of proteins involved in growth and regulation [169], which are the basis for its therapeutic properties. Its anti-inflammatory and immunomodulatory effects are mediated by a decreased number of macrophages and activated dendritic cells leading to decreases in TNF-α [170]; IL-4, IL-1beta, and IL-12p40; and nitric oxide synthesis [171,172,173]. Alitretinoin’s anti-proliferative and apoptotic effects are associated with the downregulation of RAR- and RXR-mediated receptors. Upon binding to these receptors, it downregulates the expression of IL-6 receptors and reduces the expression of viral-encoded oncogenes (Figure 6) that contribute to the lesions of Kaposi’s sarcoma [174].

### 6.2. Second-Generation Retinoids

Etretinate is a highly lipophilic enoate and ethyl ester with a prolonged half-life of approximately 120 days. Due to its strong affinity for adipose tissue, it can persist in the body for up to three years after discontinuation [175,176]. Thus, its use has been largely discontinued due to its long-acting teratogenic effects, with detectable plasma levels even years post-therapy. However, a 25-year retrospective analysis reported the delivery of a healthy baby just one year after etretinate cessation [177]. Acitretin, the primary active metabolite of etretinate, differs structurally from etretinate by replacing the cyclohexenyl group with a 4-methoxy-2,3,6-trimethylphenyl group while retaining the all-trans-tetraene structure of the retinoic acid side chain. Compared with etretinate, acitretin is more water-soluble and demonstrates minimal accumulation in adipose tissue, reducing its long-term retention [178]. Acitretin competes with RA for retinoic acid-binding protein and can activate—though not directly bind to—all RAR and RXR subtypes [179,180]. It exerts anti-inflammatory and anti-proliferative effects while normalizing keratinocyte differentiation in the epithelium. Additionally, acitretin inhibits the expression of proinflammatory cytokines, including IL-6, migration inhibitory factor-related protein-8 (MRP-8), and interferon-γ, contributing to its therapeutic efficacy in inflammatory skin conditions [181]. Acitretin is a metabolite of etretinate; however, it can undergo reverse metabolism, converting back into etretinate, together with prolonged drug persistence in the body in a process enhanced by alcohol intake [182]. Recently, Jeong et al. [183] showed that the serum levels of acitretin, but not etretinate, decrease the longer the drug has been discontinued. Additionally, higher serum acitretin concentrations were observed in older individuals. Contrary to previous findings suggesting that alcohol consumption enhances the conversion of acitretin into etretinate, alcohol was found to significantly affect serum etretinate levels. Interestingly, the frequent consumption of vitamin A or provitamin A-rich foods and supplements was linked to increased serum acitretin levels, while less frequent intake correlated with higher serum etretinate levels in patients taking acitretin. Acitretin is an FDA-approved monotherapy for various forms of psoriasis (Figure 6), including severe plaque-type psoriasis, generalized pustular psoriasis, and localized pustular psoriasis [184]. Beyond psoriasis, it is also used to treat Darier’s disease, pityriasis rubra pilaris, and lamellar ichthyosis. Additionally, acitretin has shown therapeutic potential in managing conditions such as Grover’s disease (transient acantholytic dermatosis), lichen planus, and lupus erythematosus [185].

## 7. Conclusions

Retinoids and carotenoids are vital compounds with essential roles in human health, particularly in cellular protection, skin health, and the management of various medical conditions. Retinol, as a crucial vitamin, must be acquired from the diet, either in preformed forms such as retinyl esters or as provitamin A carotenoids, which are essential for maintaining proper vision, immune function, and skin health. While ATRA plays an important biological role, its presence in the body is limited, as it is primarily metabolized, rather than stored. This underscores the importance of maintaining an adequate dietary intake of vitamin A in both its preformed and provitamin carotenoid forms.

The nutritional value of carotenoids is highly dependent on the balance between trans- and cis-isomers in food sources, with food processing influencing their bioactivity. One of the key functions of both retinoids and carotenoids is their antioxidant activity, which protects cells from oxidative damage caused by photo-oxidation and ROS.

These compounds are adept at quenching singlet oxygen, preventing cellular damage, and stabilizing free radicals formed during oxidative stress. Carotenoid oxidization leads to various metabolites, including epoxides, hydroxyketones, and peroxyl radicals, which paradoxically increase the demand for antioxidant defenses. The interaction between carotenoids and LOXs also plays a significant role in regulating oxidative stress, although this can be a double-edged sword, as LOX co-oxidation may reduce the antioxidant efficacy of carotenoids. These interactions between retinoids, carotenoids, and oxidative species underscore their protective role in maintaining cellular integrity and influencing metabolic pathways.

Both first- and second-generation retinoids have proven invaluable in treating various dermatological conditions, particularly in addressing acne, photoaging, psoriasis, and other skin disorders. First-generation retinoids, such as tretinoin, isotretinoin, and alitretinoin, bind retinoic acid receptors, thus regulating the expression of genes responsible for cell differentiation, proliferation, and apoptosis. Tretinoin, for instance, has demonstrated efficacy in treating acne and photoaging through enhancing skin structure and rejuvenation, while isotretinoin remains a powerful systemic treatment for severe acne. Alitretinoin’s immune-modulatory properties are particularly important in treating conditions such as Kaposi’s sarcoma, highlighting its oncological relevance. Additionally, ATRA is a cornerstone in the treatment of acute promyelocytic leukemia, where it promotes the differentiation of malignant promyelocytes and induces remission. Second-generation retinoids, such as etretinate and acitretin, offer pharmacological advantages, including improved water solubility and reduced adipose tissue retention, making them preferable for treating chronic conditions such as psoriasis. Beyond dermatology, retinoids’ ability to modulate cell growth and induce apoptosis has also been explored in the chemoprevention and treatment of other malignancies, including certain skin cancers and head and neck cancers. Despite these benefits, the long-term retention and teratogenic risks associated with some of these compounds require careful patient management. Nevertheless, the therapeutic benefits of these retinoids, when monitored appropriately, continue to be of great value not only in dermatology but also in oncology.

The future of retinoid therapy holds significant promise, with ongoing research focused on improving the safety, precision, and effectiveness of these compounds. Emerging strategies include the development of novel retinoids with selective affinity for RARγ, which predominates in skin tissue and offers a promising target for minimizing systemic side effects while enhancing therapeutic outcomes. Additionally, advances in nanotechnology-based delivery systems—such as nanoparticles, liposomes, and micelles—aim to enhance the bioavailability and tissue-specific delivery of retinoids, potentially overcoming limitations related to poor solubility and systemic toxicity. As new retinoid formulations and delivery systems emerge, their role in both dermatological and systemic disease management will likely only expand. The continued development of retinoid therapies offers the potential for more targeted treatments, addressing specific patient needs while minimizing the risk of side effects. In this way, retinoids will remain a cornerstone of both skin health and the treatment of various systemic conditions, contributing to improved patient outcomes and quality of life.

## Data Availability

Not applicable.

## Abbreviations

The following abbreviations are used in this manuscript:

ARAT	acyl-CoA acyl transferase
ATRA	all-trans-retinoic acid
BCMO1	β-carotene-15,15′-dioxygenase
BCDO2	β-carotene-9′,10′-dioxygenase
CD36	Cluster Determinant 36
CEL	carboxyl ester lipase
CRBP	cellular retinol-binding protein
HDL	High-Density Lipoprotein
LOX	lipoxygenase
LDL	Low-Density Lipoprotein
LP	pancreatic lipase
LRAT	lecithin retinol acyl transferase
MRP-8	migration inhibitory factor-related protein-8
PGHS	prostaglandin H synthase
RAR	retinoic acid receptor
RARE	retinoic acid response element
ROS	reactive oxygen species
RBP	retinol-binding protein
RXR	retinoid X receptor
STRA6	STimulated by Retinoic Acid 6
VLDL	Very Low-Density Lipoprotein
